# Involvement of sweet pepper *CaLOX2* in jasmonate‐dependent induced defence against Western flower thrips

**DOI:** 10.1111/jipb.12742

**Published:** 2019-02-27

**Authors:** Sandeep J Sarde, Klaas Bouwmeester, Jhon Venegas‐Molina, Anja David, Wilhelm Boland, Marcel Dicke

**Affiliations:** ^1^ Laboratory of Entomology Wageningen University P.O. Box 16 6700 AA Wageningen The Netherlands; ^2^ Laboratory of Phytopathology Wageningen University P.O. Box 16 6700 AA, Wageningen The Netherlands; ^3^ Department of Bioorganic Chemistry Max Planck Institute for Chemical Ecology Hans‐Knöll‐Straße 8 D‐07745 Jena Germany

## Abstract

Insect herbivory can seriously hinder plant performance and reduce crop yield. Thrips are minute cell‐content‐feeding insects that are important vectors of viral plant pathogens, and are serious crop pests. We investigated the role of a sweet pepper (*Capsicum annuum*) lipoxygenase gene, *CaLOX2,* in the defense of pepper plants against Western flower thrips (*Frankliniella occidentalis*). This was done through a combination of *in‐silico,* transcriptional, behavioral and chemical analyses. Our data show that *CaLOX2* is involved in jasmonic acid (JA) biosynthesis and mediates plant resistance. Expression of the JA‐related marker genes, *CaLOX2* and *CaPIN II*, was induced by thrips feeding. Silencing of *CaLOX2* in pepper plants through virus‐induced gene silencing (VIGS) resulted in low levels of *CaLOX2* transcripts, as well as significant reduction in the accumulation of JA, and its derivatives, upon thrips feeding compared to control plants. *CaLOX2*‐silenced pepper plants exhibited enhanced susceptibility to thrips. This indicates that *CaLOX2* mediates JA‐dependent signaling, resulting in defense against thrips. Furthermore, exogenous application of JA to pepper plants increased plant resistance to thrips, constrained thrips population development and made plants less attractive to thrips. Thus, a multidisciplinary approach shows that an intact lipoxygenase pathway mediates various components of sweet pepper defense against *F. occidentalis*.

## Graphical abstract

Through a combination of *in‐silico*, transcriptional, behavioural and chemical analyses, here we demonstrate that the lipoxygenase gene, *CaLOX*2, mediates jasmonate‐dependent induced defence of sweet pepper plants against Western flower thrips.

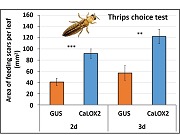

**Edited by:** Pradeep Kachroo, University of Kentucky, USA

## INTRODUCTION

In nature, land plants and insects have coexisted for more than 400 million years. Plants perceive herbivorous insects by the specific pattern of tissue disruption and/or chemical cues originating from insects (Bonaventure 2012; Heidel‐Fischer et al. 2014). Plants have three main signal‐transduction pathways, each involving a major plant hormone; i.e., jasmonic acid (JA), salicyclic acid (SA) and ethylene (ET), underlying induced defence against attackers such as herbivorous insects (Pieterse et al. 2012; Stam et al. 2014). JA is well‐known to be a key regulator of defense, induced by chewing insects and cell‐content feeders like thrips (De Vos et al. 2005; Abe et al. 2008; Abe et al. 2009; Pieterse et al. 2012), whereas SA is known to mediate induced plant defense responses against phloem feeders (Zhu‐Salzman et al. 2004; Walling 2008; Pieterse et al. 2012; Tzin et al. 2015). The three major signaling pathways may exhibit crosstalk. For instance, JA and SA usually act antagonistically, but are also reported to act synergistically, or additively (Pieterse et al. 2012; Thaler et al. 2012).

The Western flower thrips, *Frankliniella occidentalis* (Pergande) (Thysanoptera: Thripidae) is a polyphagous and economically important pest. Thrips insert their stylets into plant tissues and ingest cell contents, resulting in a silvery appearance of the damaged area (Steenbergen et al. 2018). They feed on almost all aboveground organs of pepper plants and are considered the most devastating pest in greenhouses, worldwide. Their feeding affects leaf size and photosynthetic capacity, which eventually reduces plant growth and productivity (Steiner 1990; Welter et al. 1990; Shipp et al. 1998). Thrips often aggregate in narrow crevices on the plants, such as in the flowers, developing fruits, foliage and buds, making them difficult to control. Moreover, they also cause indirect damage by transmitting tospoviruses, such as *Tomato spotted wilt virus* (TSWV) (Maris et al. 2003). Thus, the development of novel approaches to control thrips damage by using knowledge on the molecular mechanisms of plant responses is important.

Various studies have addressed induced plant defenses against leaf‐chewing and phloem feeding herbivores (Walling 2000; Bonaventure 2012; Heidel‐Fischer et al. 2014; Stam et al. 2014; Zust and Agrawal 2016), whereas much less is known about plant responses to cell‐content feeding thrips. To our knowledge, few studies have reported on the role of JA in regulating induced plant defense responses against thrips feeding. In *Arabidopsis* and Chinese cabbage (*Brassica rapa* subsp*. pekinensis*), JA is involved in defense against thrips (Abe et al. 2008; Abe et al. 2009). In tomato, the JA‐signaling mutant, *Defenceless1* (*Def1)*, exhibits enhanced susceptibility to thrips feeding (Li et al. 2002; Escobar‐Bravo et al. 2017) and a decline in thrips abundance was observed in tomato upon JA application in field conditions (Thaler et al. 2001).

Likewise, cotton and soybean plants also show increased resistance against thrips upon exogenous JA or MeJA application (Omer et al. 2001; Selig et al. 2016). In *Arabidopsis,* a total of 199 genes were differentially expressed (up and downregulated) upon thrips feeding, among which 138 (69%) were JA‐responsive genes (De Vos et al. 2005). However, in pepper plants, little is known about the mechanisms underlying defense against insect herbivores.

Lipoxygenases (LOXs) are enzymes encoded by a multi‐gene family functioning in different plant developmental and defense processes (Brash 1999). They are well‐known to oxygenate fatty acids. In plants, the main classes are 9‐lipoxygenases and 13‐lipoxygenases that oxygenate lipids at the 9^th^ and 13^th^ carbon atom, respectively (Feussner and Wasternack 2002). In JA biosynthesis, the first oxygenation step of linolenic acid is performed by a 13‐lipoxygenase (Brash 1999; Feussner and Wasternack 2002). Disruption of this 13‐LOX has been shown to suppress the JA pathway in several plant species, resulting in enhanced susceptibility to insect herbivory.

In *Nicotiana attenuata* silencing through antisense expression of *NaLOX3*, involved in JA synthesis, suppressed JA synthesis and enhanced herbivore performance (Halitschke and Baldwin 2003). Similarly, in tomato, overexpression of *TomLOXD* elevated levels of JA and resistance to a caterpillar species (Yan et al. 2013). The 13‐LOXs have also been identified in *Arabidopsis* (Bell et al. 1995), potato (Royo et al. 1996), rice (Zhou et al. 2009) and Asian ginseng (Rahimi et al. 2017). In pepper, a 9‐LOX, *CaLOX1,* has been identified and reported to be involved in defense and cell‐death responses to pathogens (Hwang and Hwang 2010). However, to date, no 13‐type LOX has been characterized in pepper (*Capsicum annuum*).

The main objective of the present study was to identify and characterize a 13‐*LOX* gene specifically involved in JA biosynthesis and subsequently elucidate its role in JA‐regulated defense of a non‐model plant, sweet pepper, against thrips. We performed *in‐silico* analysis to identify the pepper 13‐lipoxygenase gene that is potentially involved in JA‐biosynthesis (termed *CaLOX2*) and evaluated performance and preference of thrips on plants subjected to exogenous application of JA and silencing of the *CaLOX2* gene by virus‐induced gene silencing (VIGS). For this, we initially investigated the induction of JA‐ and SA‐regulated genes, upon thrips feeding, and long‐term effects of exogenous JA application on thrips population size, preference of thrips between JA‐treated and non‐treated plants, and plant resistance. Furthermore, the consequences of silencing *CaLOX2* through VIGS for production of down‐stream JA‐related phytohormones and performance and preference of thrips were studied. Thus, this study assesses the role of *CaLOX2* in jasmonic acid‐dependent signaling underlying the defense response to thrips feeding in the non‐model plant sweet pepper.

## RESULTS

### Identification of CaLOX2 in pepper

To identify the *LOX2* homolog in pepper, all sequences of tomato LOX proteins were used as queries in blast searches for a genome‐wide search against the protein database of *Capsicum annuum L. Zunla‐1*. This resulted in the identification of several LOX proteins in pepper (Sarde et al. 2018). These LOX homologs were further scanned for the presence of PLAT/LH2 and LOX domains that are hallmarks of the lipoxygenase gene family (Chen et al. 2015), using the Pfam database. To narrow down the selection towards the specific LOX protein induced upon herbivory or wounding in sweet pepper, the closest homolog of the tomato *LOXD* gene, well‐known to have a similar function (Yan et al. 2013), was selected from pepper (*CaLOX2, Capana03g000103*) and further subjected to phylogenetic and synteny analysis.

**Figure 1:**
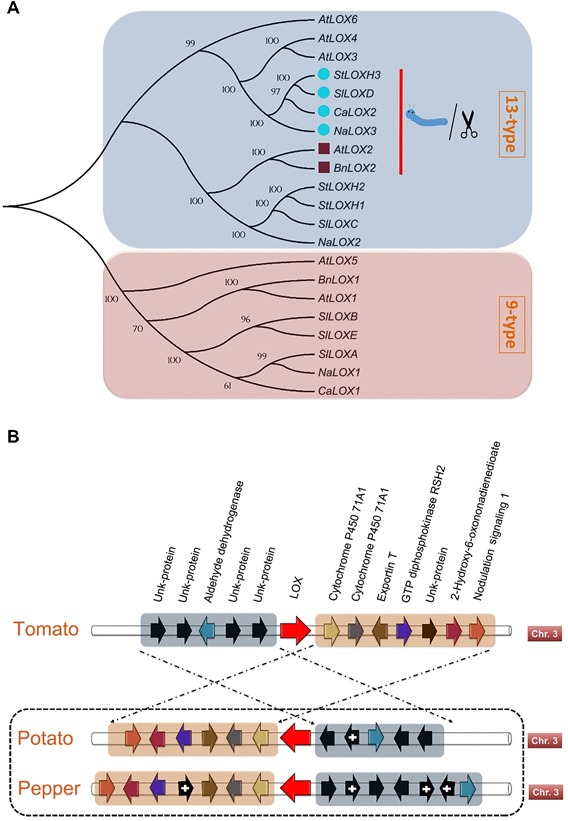
**Phylogenetic and synteny analyses of lipoxygenases** (**A**) Phylogenetic relationship of lipoxygenases of six species from the Brassicaceae and Solanaceae families. The tree was generated by MEGA 5.1 using the Maximum Likelihood method with 1,000 bootstraps. Accession numbers or Gene IDs of sequences used to construct the phylogenetic tree are as follows. *Arabidopsis thaliana: AtLOX1,* AAA32827*; AtLOX2,* AAA32749*; AtLOX3,* AT1G17420*; AtLOX4,* AT1G72520*; AtLOX5,* AT3G22400*; AtLOX6,* AT1G67560*; Brassica napus: BnLOX1,* AAO03558*, BnLOX2,* NP_001303054*; Solanum lycopersicum: SlLOXC,* AAB65766*; SlLOXD,* AAB65767; *SlLOXB,* AAA53183*; SlLOXE,* AAG21691*; SlLOXA,* AAA53184; *Solanum tuberosum: StLOXH2,* CAA65268*; StLOXH3,* CAA65269*; StLOX1,* AAB67858*; Capsicum annuum: CaLOX2,* Capana03g000103; *CaLOX1,* ACO57136*; Nicotiana attenuata: NaLOX1,* AAP83134*; NaLOX2,* AAP83137*; NaLOX3,* AAP83138. (**B**) Syntenic organization of tomato (*SlLOXD*), potato (*StLOXH3*) and pepper (*CaLOX2*) *LOX* genes. Black arrows with and without “+” sign depict genes that are not identical to synteny of tomato or unknown (uncharacterized) genes, respectively.

Phylogenetic analysis was conducted to analyze the evolutionary relationship between several LOX proteins across different species of the Brassicaceae and Solanaceae families. The phylogenetic tree (Figure 1A) clearly represents two major clades of 13‐type and 9‐type LOX proteins. In the 13‐type clade, the related pepper LOX2 protein is positioned in the sub‐clade of LOX proteins within the Solanaceae family that are known to be involved in JA biosynthesis. Similarly, this sub‐clade appears closer to the Brassicaceae sub‐clade comprising LOX proteins that have a similar function (Figure 1A), suggesting a role of *CaLOX2* in JA biosynthesis.

Furthermore, genomic locations and assessments of syntenic maps offer insights into the conservation of genes across organisms. In the model plant of the Solanaceae family, tomato, the *TomLOXD* gene, well‐known to be induced upon herbivory, is flanked by five genes on one side and seven genes on the other side (Figure 1B). The synteny is similar, but in reverse order with some exceptions of additional, uncharacterized or unknown genes for its counterpart *LOX* genes in potato (*LOXH3*) and pepper (*CaLOX2*). Moreover, localization of this gene is still intact on Chromosome 3 in pepper, tomato and potato. Taken together the *in‐silico* analysis suggests a possible role of *CaLOX2* in the octadecanoid pathway.

### JA and SA‐related marker genes are up‐regulated upon thrips feeding

**Figure 2:**
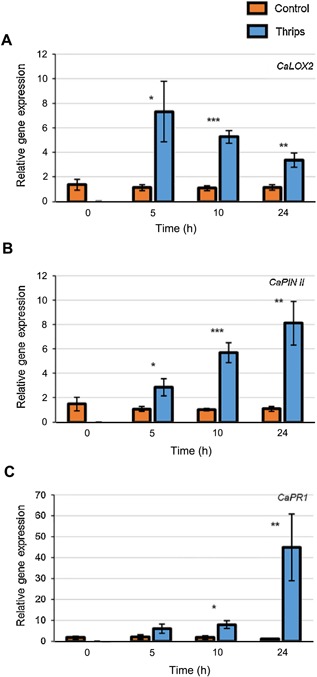
**Quantitative RT‐PCR (RT‐qPCR) of JA and SA‐related marker genes in sweet pepper leaves in response to thrips (*F. occidentalis*) feeding** **(A)** *CaLOX2* and **(B)** *CaPIN II*, as marker genes for the JA‐pathway and **(C)** *CaPR1*, as marker gene for the SA‐pathway. Five 2^nd^ instar thrips larvae fed locally (confined in clip cages) on the first true leaf of four‐week‐old pepper plants. Empty clip cages were used on control plants. The expression level of each gene was normalized to the expression of the housekeeping gene *CaACTIN*. Bars represent mean ± *SE* (*n* = 4–5 biological replicates). Bars marked with asterisks indicate significant differences (Student's *t*‐test), *P‐value < 0.05, **P‐value < 0.01, ***P‐value < 0.001.

We analyzed the expression of JA and SA‐related marker genes in sweet pepper in response to thrips feeding in order to determine the role of JA in defense against thrips. Expression of the *in‐silico* identified *CaLOX2* gene was induced upon thrips feeding at all three time points sampled (Figure 2A). Similarly, expression of the downstream JA‐responsive gene, *CaPIN II,* was also up‐regulated upon thrips feeding (Figure 2B). Moreover, upon thrips infestation the SA‐responsive gene, *CaPR1*, did not show significant induction after 5h but was upregulated after 10 and 24 h of thrips feeding (Figure 2C).

### Exogenous JA application negatively affects thrips feeding and preference

**Figure 3:**
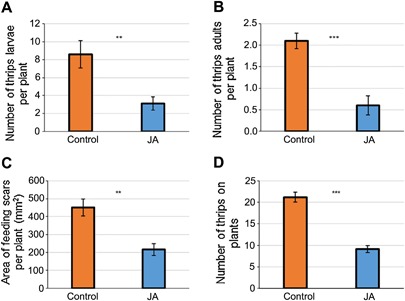
**No‐choice (thrips population and feeding damage) and choice (preference) tests of Western flower thrips upon exogenous application of JA (100 µM)** **(A, B)** Effect of JA application on thrips population and **(C)** feeding damage. Twenty‐five adult females fed on 4‐week‐old sweet pepper plants for 2 weeks. Water + Tween20 (control) or 100 µM JA + Tween20 (treatment) was applied 1 d before thrips were introduced on pepper plants. Number of thrips larvae **(A)**, thrips adults **(B)** and feeding damage **(C)** in mm^2^ caused by thrips feeding on plants was assessed after two‐weeks. Mean ± *SE* based on 10 and 12 biological replicates for thrips population and feeding damage, respectively. Asterisks indicate significant differences (Student's *t*‐test), **P‐value < 0.01, ***P‐value < 0.001. **(D)** Effect of exogenous JA on host plant preference of thrips (choice experiment). Mean ± *SE* based on eleven biological replicates. Asterisks indicate significant differences (Student's *t* test, *t* = 7.598); ***P‐value < 0.001.

Because thrips feeding induces the transcription of JA‐related genes, we first investigated the effect of exogenous JA application on thrips population build‐up and its feeding. For this purpose, 25 adult females were introduced onto control and JA‐treated plants. After two weeks, the larvae and adults were counted on each plant. The JA‐treated plants had significantly fewer larvae and adult thrips compared to control plants (Figure 3A, B). Control plants had 2.8 times more larvae (first and second instars; Figure 3A) and 3.5 times more adults (Figure 3B) than JA‐treated plants. This indicates that JA underlies pepper defense against thrips.

We subsequently investigated the effect of exogenous JA application on thrips feeding, in the same experimental setup. Significantly more feeding damage was recorded on control plants (450 mm^2^) compared to JA‐treated plants (216 mm^2^) (Figure 3C). These results further support the involvement of JA in resistance of pepper against thrips.

Finally, we investigated the effect of JA on host plant preference of thrips. To this end, we placed 50 adult females halfway between a control and a JA‐treated plant and counted the number of thrips on each plant after 2 d; thrips significantly preferred control plants over JA‐treated plants (Figure 3D).

### VIGS of CaLOX2 suppresses CaLOX2 expression

To study the involvement of *CaLOX2* in the JA pathway, we silenced *CaLOX2* in pepper plants using VIGS. The unique region of 282 bp of the *CaLOX2* coding region (File S1) was selected, using CLC bio‐workbench and its specificity was confirmed using the VIGS tool from the Sol Genomics Network.

**Figure 4:**
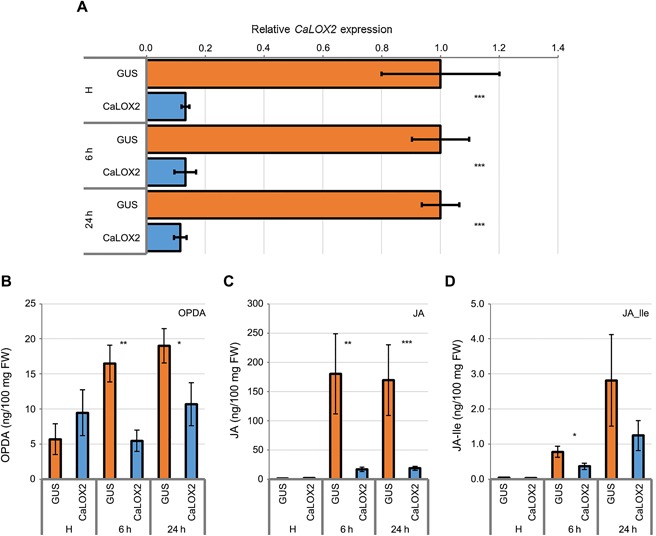
**Quantitative RT‐PCR (RT‐qPCR) of *CaLOX2* and phytohormone quantification in GUS‐vector control and *CaLOX2*‐silenced leaves infested with western flower thrips for 6 h and 24 h** **(A)** Silencing efficiency of *CaLOX2*. H, healthy leaves; GUS, β‐Glucuronidase; *CaLOX2, C. annuum Lipoxygenase2*. The *C. annuum* actin gene was used for normalization in qPCR. Data are mean ± *SE* of 14 biological replicates from two independent experiments. Asterisks indicate significant differences (Student's t test), ***P‐value < 0.001. **(B–D)** Quantification of JA and its derivatives. OPDA, 12‐oxo‐phytodienoic acid; JA, Jasmonic acid; JA‐Ile, Jasmonic acid isoleucine. Five adult females fed on a detached single leaf of a 5‐week‐old plant. Data are mean ± *SE* of 6 biological replicates. Asterisks indicate significant differences (Student's *t* test), *: *P*‐value < 0.05, **: *P*‐value < 0.01, ***: *P*‐value < 0.001.

To assess the efficiency of VIGS, *CaLOX2* transcript levels were quantified using qRT‐PCR in GUS‐vector control (TRV:*GUS*) and *CaLOX2*‐silenced (TRV:*CaLOX2*) pepper leaves infested with thrips. *CaLOX2* expression was significantly induced, at both time points, in GUS‐vector control leaves (Figure S1). In contrast, in *CaLOX2*‐silenced pepper leaves, *CaLOX2* transcript levels were significantly decreased compared to GUS‐vector control leaves, both experiencing thrips feeding, indicating that *CaLOX2* silencing was effective in suppressing *CaLOX2* induction (Figure 4A).

### Silencing of CaLOX2 compromises jasmonate accumulation

To investigate the effect of *CaLOX2* silencing on the accumulation of JA and its derivatives in response to thrips attack, we assessed the levels of phytohormones in leaves with the GUS‐vector and *CaLOX2*‐silenced pepper plants infested with thrips (Figure 4). *CaLOX2* silencing resulted in lower levels of OPDA, JA and JA‐Ile. The differences were statistically significant at 6 h for all three phytohormones and at 24 h for OPDA and JA. This indicated that *CaLOX2* silencing compromised the induction of JA, and its derivatives JA‐Ile, and OPDA in *CaLOX2*‐silenced pepper leaves subjected to thrips feeding (Figure 4B–D). These results support a role of *CaLOX2* in the octadecanoid pathway leading to the biosynthesis of JA and JA‐Ile, as induced upon herbivory.

### Suppression of CaLOX2 confers increased susceptibility to thrips feeding

**Figure 5:**
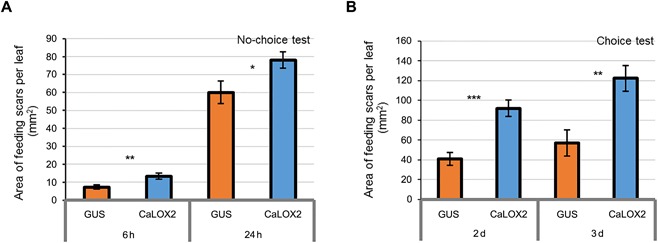
**Effect of *CaLOX2* silencing by VIGS in sweet pepper plants on thrips feeding in no‐choice and thrips preference in choice tests** (**A**) Area of feeding scars per leaf after 6 and 24 h of thrips feeding (five female thrips adults per leaf) in no‐choice assay. Mean ± *SE,* based on fourteen biological replicates from two independent experiments. Asterisks indicate significant differences (Student's *t*‐test), *P‐value < 0.05, **P‐value < 0.01. (**B**) Area of feeding scars per leaf after 2 and 3 d of infestation by five female thrips adults in choice assay. Mean ± *SE*, based on 14 biological replicates from two independent experiments. Asterisks indicate significant differences (paired *t*‐test), **P‐value < 0.01, ***P‐value < 0.001.

To assess the consequence of *CaLOX2* silencing for thrips feeding and preference, we quantified thrips feeding damage on leaves of GUS‐vector control and *CaLOX2*‐silenced pepper plants. This was done in no‐choice (Figure 5A) and choice (Figure 5B) tests. Injury resulting from thrips attack was significantly greater on *CaLOX2*‐silenced plants than on non‐silenced (GUS) plants, in a no‐choice test, at both time points, indicating that a functional *CaLOX2* reduces the feeding rate of thrips individuals (Figure 5A). Furthermore, when provided with a choice between silenced leaves (*CaLOX2‐*silenced) and non‐silenced (GUS‐vector) leaves, thrips clearly preferred to feed on *CaLOX2*‐silenced plants (Figure 5B). Taken together, these results support the role of *CaLOX2* in the octadecanoid pathway of induced defense.

## DISCUSSION

Through a combination of an *in‐silico* analysis and transcriptional, behavioral and chemical analyses, we show that *CaLOX2* (Capana03g000103) is activated by thrips feeding, and involved in the octadecanoid pathway, leading to the phytohormone JA and its active conjugate JA‐Ile, resulting in induced defense against thrips in sweet pepper. Although jasmonates have been reported as important players underlying induced plant defense against various herbivorous insect species (Wasternack 2007; Howe and Jander 2008), most research has focused on defenses against leaf‐chewing or phloem‐feeding herbivores (De Vos et al. 2005; Bonaventure 2012; Heidel‐Fischer et al. 2014; Stam et al. 2014; Zust and Agrawal 2016).

By contrast, relatively little is known about induced plant defenses in response to cell‐content feeders like thrips. Most knowledge on inducible defense against thrips is available for model plants, such as *Arabidopsis thaliana* and tomato, for which specific well‐characterized mutants are available (Li et al. 2002; Abe et al. 2008; Abe et al. 2009; Abe et al. 2012). Disentangling the molecular mechanisms underlying induced defense against thrips in crops will be valuable in the selection of resistant varieties.

Few studies have reported that jasmonates regulate induced defenses against thrips (Li et al. 2002; Abe et al. 2008; Abe et al. 2009). Due to the relatively limited availability of genetic tools and genomic information, little is known about the biosynthesis of JA and the role of jasmonates in modulating defense responses in non‐model plants, such as pepper. Nonetheless, recent knowledge on genomic information and valuable tools like VIGS provides valuable resources to investigate the role of the octadecanoid pathway in defense against thrips feeding. We used these tools to identify *CaLOX2* as being the *LOX* gene involved in the JA‐pathway and resistance of cultivated pepper to *F. occidentalis*.

By *in‐silico* analysis, we identified *CaLOX2* as the gene involved in the octadecanoid pathway. *CaLOX2* is a 13‐type LOX that is phylogenetically close to functionally similar orthologues in tomato (*SlLOXD*), potato (*StLOXH3*) and tobacco (*NaLOX3*), which are specifically involved in the biosynthesis of JA (Figure 1A) (Royo et al. 1996; Kessler et al. 2004; Yan et al. 2013). Moreover, chromosomal locations and comparisons of syntenic maps show that flanking genes are conserved, with some exceptions of uncharacterized or additional genes, and that the *CaLOX2* gene is positioned on Chromosome number 3, across selected plants from the Solanaceae family. This supports the evolutionary significance of this gene to the plants and supports a role of *CaLOX2* in the octadecanoid pathway, as further reinforced experimentally in our study.

Our findings clearly show that the JA‐related genes, *CaLOX2* and *CaPIN II*, are upregulated at all time points upon thrips feeding, as also recorded for respective JA‐related genes in *Arabidopsis* and Chinese cabbage (*Brassica rapa subsp. pekinensis*) (De Vos et al. 2005; Abe et al. 2008; Abe et al. 2009). Moreover, the upregulation of *LOX* genes was also recorded in cabbage (*Brassica oleracea* L.*)* and tomato, upon feeding by another cell‐content feeding herbivore, the spider mite *Tetranychus urticae* (Li et al. 2002; Zheng et al. 2007). In contrast, no upregulation of *CaPR1* (SA‐related) was recorded until 10 and 24 h after initiation of thrips feeding, similar to what has been recorded for the SA‐related genes *PR1* and *BGL.2* in *Arabidopsis* (Abe et al. 2008). De Vos et al. (2005) reported that SA levels were elevated after 12 h of thrips feeding, corresponding with induction of SA‐related genes at later time points. Possibly, thrips manipulate plant defense by inducing SA, to stimulate antagonistic crosstalk with the JA pathway to interfere with plant defense (Abe et al. 2012; Stam et al. 2014).

Both exogenous application of JA and silencing of *CaLOX2,* which interferes with JA induction demonstrate that JA is involved in inducible defense against thrips. JA is also involved in inducible defense against another cell‐content feeding herbivore, the spider mite *Tetranychus urticae* in Lima bean and tomato plants (Dicke et al. 1999; Li et al. 2002; Gols et al. 2003; Ament et al. 2004). Similar induction of resistance against thrips, by the application of exogenous JA, was also recorded in *A. thaliana*, *Brassica rapa* and *S. lycopersicum* (Li et al. 2002; Abe et al. 2008; Abe et al. 2009).

There are several homologs of *LOX* genes in a wide range of plants (Zhang et al. 2006; Podolyan et al. 2010; Liu et al. 2011; Umate 2011; Zhang et al. 2014; Chen et al. 2015). They have been thoroughly characterized and reported to be involved in several plant biological processes, such as plant defense, tuber growth, germination of seeds, and fruit ripening (Kolomiets et al. 2001; Bailly et al. 2002; Porta and Rocha‐Sosa 2002; Barry and Giovannoni 2007; Abe et al. 2008).

Usually, there is at least one *LOX* homolog induced upon herbivory and involved in the biosynthesis of JA (Kessler et al. 2004; Zhou et al. 2009; Allmann et al. 2010; Zheng et al. 2011; Yan et al. 2013). These phenomena have been well studied in model plants, but little is known in non‐model plants. In order to study the role of octadecanoid pathway in different biological processes in plants, JA‐deficient mutants have been generated. In *Arabidopsis*, the *Coi1*‐mutant is often used; this mutant is defective in the JA‐Ile receptor (Xie et al. 1998). In tomato, several mutant lines, including *Def‐1*, *Spr‐1*, and *Spr‐2*, are available (Howe et al. 1996; Howe and Ryan 1999; Li et al. 2002).

Nonetheless, the genomic target region of JA‐deficient mutants in tomato is still unclear. In rice and tobacco, a specific homolog of *LOX* was targeted to generate JA‐deficient plants through transformation (Kessler et al. 2004; Zhou et al. 2009). Silencing of *OsHI‐LOX* or *Na‐LOX3* in rice and tobacco, respectively, interfered with the induction of JA upon herbivore feeding (Halitschke and Baldwin 2003; Zhou et al. 2009; Lu et al. 2015). Likewise, in a recent study in barley, overexpression and downregulation of *LOX2.2* affected the expression of JA‐related genes depicting its role in JA‐biosynthesis (Losvik et al. 2017).

The present study used gene silencing of *CaLOX2*, instead of transformation, and demonstrates that *CaLOX2* interferes with the production of not only JA and but also other jasmonates, such as OPDA and JA‐Ile, upon thrips feeding on pepper, underlining its role in the octadecanoid pathway. Moreover, our data show that targeting a specific 13‐type *LOX* gene in a non‐model plant can be effective to suppress the entire jasmonate cascade.

Silencing *CaLOX2* clearly resulted in enhanced feeding and preference of thrips for *CaLOX2*‐silenced leaves, compared to control leaves. Furthermore, in *Nicotiana attenuata* and rice, JA‐silenced plants were more vulnerable to herbivorous insects (Kessler et al. 2004; Zhou et al. 2009). Moreover, silencing *LOX3* in tobacco influenced herbivore community composition under field conditions (Kessler et al. 2004). Such effects are likely the consequence of altered plant phenotype in terms of secondary metabolites or proteinase inhibitors whose biosynthesis is modulated by JA, differentially affecting different members of the plant‐associated insect community.

The possibility exists that JA also regulates the defense of pepper, indirectly, by influencing herbivore‐induced plant volatiles, thereby mediating the attraction of carnivorous arthropods, such as parasitoids or predators of thrips, since the importance of JA in attraction of carnivorous enemies of herbivores has been reported for several plant species (Dicke et al. 1999; Gols et al. 2003; Halitschke et al. 2008; Ozawa et al. 2008). The *CaLOX2‐*silenced plants will be useful to investigate the role of this gene in indirect defense of sweet pepper.

## MATERIALS AND METHODS

### Plant material and thrips rearing

Sweet pepper (*Capsicum annuum*; Mandy variety) (Rijk Zwaan, De Lier, The Netherlands) plants were grown in pots of 12 cm diameter in a greenhouse at 23–25°C with a 16L:8D photoperiod and 70% ±10% relative humidity. Four‐week‐old plants were used in the experiments. One day before the experiments, plants were transferred to a climate chamber with controlled conditions (24 ± 1°C, 70% ± 10% relative humidity, 16L:8D photoperiod and light intensity of 70 µmol photons m^−2^ s^−1^). Western flower thrips (WFT; *Frankliniella occidentalis*) were reared on bean pods (*Phaseolus vulgaris*) in a climate‐controlled cabinet (25 ± 2°C, 70% ± 10% relative humidity, L16:8D photoperiod).

### Sequence retrieval, homology search and domain analysis

Tomato (*Solanum lycopersicum*) LOX protein sequences were retrieved from the Ensembl Plants genome browser (http://plants.ensembl.org/index.html). Sequences of LOX proteins from other plant species, i.e., *Solanum tuberosum*, *Nicotiana attenuata, Brassica napus,* and *Arabidopsis thaliana* were downloaded from the NCBI repository. The tomato LOXD (*Solyc03g122340*) protein sequence was used to identify *CaLOX2* in pepper. All LOX proteins were subjected to Pfam (v27.0) domain analysis (Finn et al. 2014) using CLC Main Workbench (Version 7.6.4). The pepper genome/proteome subjected to analysis was derived from *Capsicum annuum L. Zunla‐1* (Qin et al. 2014).

### Phylogenetic and synteny analysis

Full‐length protein sequences were used for alignment with the MUSCLE tool (https://www.ebi.ac.uk/Tools/msa/muscle/) using the default parameters. The obtained alignment was used for construction of a Maximum Likelihood phylogenetic tree with 1,000 bootstrap replicates using MEGA5.1 (Tamura et al. 2011). Synteny analysis of solanaceous *LOX* genes, specifically involved in the octadecanoid pathway, was performed using the Ensembl Plants genome browser (Yates et al. 2016), Mapviewer from NCBI (https://www.ncbi.nlm.nih.gov/mapview/) and the Gene database from NCBI (https://www.ncbi.nlm.nih.gov/gene).

### Plasmid construction

The pTRV (*Tobacco rattle virus*)‐based VIGS protocol (Wang et al. 2013; Senthil‐Kumar and Mysore 2014) was used to generate *CaLOX2‐*silenced (TRV:*CaLOX2*) pepper plants. For this purpose, the unique coding gene fragment of the *Lipoxygenase‐2* gene of *Capsicum annuum* (*CaLOX2, Capana03g000103*) was selected and amplified by PCR. The specificity of the selected sequence was checked via the VIGS tool on the Sol Genomics Network (http://vigs.solgenomics.net/). A gene fragment of 282 bp (Supplementary File S1) was cloned into the TRV2‐vector and subsequently transformed into *Agrobacterium tumefaciens* GV3101 strain via electroporation. Presence of the *CaLOX2* fragment in the TRV2‐vector was verified by restriction digestion and sequencing.

### Agro‐infiltration and TRV‐mediated silencing assays

*Agrobacterium tumefaciens* GV3101 strains carrying vectors were grown overnight at 28°C in YEB (Yeast Extract Broth) media with appropriate antibiotics (rifampicin 25 μg/mL, kanamycin 50 μg/mL). *A. tumefaciens* cells were centrifuged and resuspended in induction medium containing rifampicin (25 μg/mL), kanamycin (50 μg/mL) and acetosyringone (50 μg/mL) antibiotics for 3–4 h, and thereafter for 1 h in infiltration medium with acetosyringone (150 μg/mL). The composition of induction and infiltration medium is provided in File S1. *Agrobacterium tumefaciens* GV3101 cultures carrying pTRV1 and pTRV2:*GUS* or pTRV2:*CaLOX2* or pTRV2:*NaPDS* were mixed at a ratio of 1:1 to a final OD_600_ of 1.0 and syringe‐infiltrated into cotyledons of two‐week‐old pepper seedlings (Wang et al. 2013). Due to high sequence similarity between *NaPDS* and *CaPDS*, we used pTRV2:*NaPDS* construct to monitor the proliferation of silencing in pepper plants. The plants prior and post *Agrobacterium*‐infiltration were kept in a greenhouse at 23–25°C, 70% ± 10% relative humidity and 16L:8D photoperiod. Efficiency of *CaLOX2* silencing was validated by RT‐qPCR.

### No‐choice and two‐choice feeding bioassays post VIGS

To determine the role of *CaLOX2* in sweet pepper resistance to thrips, the youngest fully‐grown true leaves of three‐week‐old Agrobacterium‐infiltrated plants were used for no‐choice and two‐choice thrips bioassays. A no‐choice experiment was conducted by using one detached leaf of pTRV2:*GUS* or pTRV2:*CaLOX2* plants. Each detached leaf was placed in a separate Petri dish (140 × 20 mm^2^). The petiole was inserted in a 1% agar solution. Five adult female thrips were placed on each leaf in the Petri dish for 6 or 24 h (with 14 replicates) and thrips feeding‐associated damage was recorded at these time points. In addition, infested leaves were sampled for qRT‐PCR and phytohormone analyses. In two‐choice assays, one leaf each of a TRV2:*CaLOX2* and a TRV2:*GUS* plant were placed adjacent to each other in a Petri dish (with 14 replicates). Five adult female thrips were placed in the center of the Petri dish, equidistant from the leaves, and were allowed to feed on the leaves for two 2–3 d. The area of feeding scars (on both abaxial and adaxial leaf sides) was measured using a light microscope and a transparent grid sheet.

### JA treatment and bioassays

To test the effect of the induction of JA signaling on sweet pepper resistance to thrips, 4‐week‐old sweet pepper plants were sprayed with 100 μM JA, or mock‐treated with water, both mixed with 0.1% of Tween20 (detailed protocol in File S1). The treatment was conducted one day before the bioassay. The experiments (non‐choice and choice assays) were performed in a climate chamber under controlled conditions (24 ± 1°C, 70% ± 10% relative humidity, 70 µmol photons m^−2^ s^−1^ light intensity and 16L:8D photoperiod). For a non‐choice feeding bioassay, 25 female adult thrips were placed on each JA‐treated and non‐treated whole pepper plants confined in transparent plastic cages covered with mesh on top. After 14 d, the number of their offspring (first and second‐instar larvae) and feeding damage on plants was assessed. The experiment was executed twice, one for assessment of offspring number (with 10 replicate plants) and one for assessment of feeding damage (with 12 replicate plants). In preference (choice) assays (with 11 replicates), a JA‐treated and a mock‐treated plant were positioned on either side of a transparent plastic box (height: 310 mm, width: 440 mm, length: 710 mm) and 50 adult female thrips were placed halfway between them. The boxes were incubated in a climate chamber with controlled conditions (24 ± 1°C, 70% ± 10% relative humidity, 16L:8D photoperiod). Two d later, the numbers of thrips on each plant were recorded.

### RNA isolation and qRT‐PCR

To test the effect of thrips feeding on JA and SA‐associated defense genes, five second instar thrips larvae (L2) were introduced into a clip cage and allowed to feed on one of the first two true leaves of four‐week‐old sweet pepper plants. Plants with clip cages without thrips served as controls. At 0, 5, 10 and 24 h after infestation, leaves were sampled for gene expression analyses of JA‐ and SA‐associated marker genes. For each treatment and time point, four to five biological replicates were analyzed, each replicate consisting of one individual plant. RNA extraction was executed using the Bioline kit, in accordance to the manufacturer's protocol. cDNA was synthesized from 1 µg of total RNA with iScript cDNA synthesis kit (Bio‐Rad). For qPCR analysis, a 25 µL reaction mixture, containing 1 µL (10 μM) of forward and reverse primers, 12.5 µL of SYBR Green Supermix (Bio‐Rad) and 5 µL cDNA, was used. The reference gene, *CaACTIN*, was used as normalizer for determining the relative expression of JA‐related genes (*CaLOX2; Capsicum annuum Lipoxygenase 2* and *CaPIN II; Capsicum annuum Protease Inhibitor II*) and an SA‐dependent gene (*CaPR1; Capsicum annuum Pathogenesis Related 1*). The following PCR conditions were used: 3 min at 95°C, followed by 40 cycles of 15 s at 95°C, and 45 s at 60°C. At the end of each qPCR, the melting curve of each gene was recorded. All primers used for qPCR pre and post VIGS are presented in File S1.

Relative gene expression was analyzed using the geometric mean of threshold cycle (Ct) values (Vandesompele et al. 2002) for the reference gene *CaACTIN* with the 2–^ΔΔCt^ method (Livak and Schmittgen 2001).

### Hormone quantification

Leaf samples (100 mg each) from pTRV2:*GUS* and pTRV2:*CaLOX2* plants were flash‐frozen in liquid nitrogen and stored at −80°C. High‐performance liquid chromatography–mass spectrometry (HPLC‐MS/MS) was used to quantify JA (jasmonic acid), JA‐Ile (jasmonic acid isoleucine) and OPDA (12‐oxo‐phytodienoic acid) content, according to the method described in Trapp et al. (2014).

### Statistical analysis

Data on gene expression and hormone content were log‐transformed prior to statistical analyses. The data on gene expression, thrips feeding, gene silencing efficiency post VIGS and hormonal quantification were all subjected to a Student's *t*‐test. Moreover, thrips two‐choice preference data were expressed as the proportion of thrips detected on either treatment. The data were analyzed by a *t*‐test within each treatment to determine if the proportion of thrips significantly differed from 0.5, as previously described (Grostal and Dicke 1999). The data of the two‐choice feeding experiment post VIGS treatment were analyzed by a paired t‐test. These analyses were performed using software IBM SPSS Statistics for Windows, version 23 (IBM Corp., Armonk, N.Y., USA). Biological replicates used for statistics each consist of one individual plant.

## CONCLUSION

This study has shown that gene silencing by VIGS is a useful method to functionally characterize candidate genes of pepper for their role in resistance to insects. Through a multidisciplinary approach, involving *in‐silico,* transcriptional, chemical, behavioral studies and bioassays, to assess plant resistance, this study identified *CaLOX2* as being involved in the JA‐biosynthetic pathway. Moreover, JA‐dependent signaling was shown to be important in defense of pepper plants to thrips. Thus, this method allows for investigating functional roles of *C. annuum* genes in plant‐insect interactions.

## AUTHOR CONTRIBUTIONS

S.J.S., K.B. and M.D. conceived and designed the experiments; S.J.S., K.B., J.V.M. and A.D. performed the experiments; S.J.S., K.B., J.V.M., A.D., W.B. and M.D. analyzed the data; S.J.S., K.B. and M.D. contributed to manuscript writing, and all authors confirm their contributions, and read and approved the final manuscript.

## Supporting information

Additional Supporting Information may be found online in the supporting information tab for this article: http://onlinelibrary.wiley.com/doi/10.1111/jipb.12742/suppinfo

**Figure S1.** Expression of *CaLOX2* in GUS‐infiltrated samples upon thrips feeding
The expression level of each gene was normalized to housekeeping gene *CaACTIN*. Data are mean ± *SE* of fourteen biological replicates from two independent experiments. Asterisks indicate significant differences (Student's t test), ***P‐value ˂ 0.001.

**File S1.** Primers used for (q)RT‐PCR, unique region of 282bp of the CaLOX2 coding region. induction medium and infiltration medium.
