# Supplementary material for: Involvement of sweet pepper CaLOX2 in jasmonate‐dependent induced defence against Western flower thrips
Source: J Integr Plant Biol. 2019 Feb 27;61(10):1085–98. doi: 10.1111/jipb.12742 (PMC6850143; doi:10.1111/jipb.12742)
Supplement: Supplementary file 1 — Figure S1. Expression of CaLOX2 in GUS‐infiltrated samples upon thrips feeding The expression level of each gene was normalized to housekeeping gene CaACTIN. Data are mean ± SE of fourteen biological replicates from two independent experiments. Asterisks indicate significant differences (Student's t test), ***P‐value ˂ 0.001. [file JIPB-61-1085-s001.pdf]

Sandeep J Sarde[1], Klaas Bouwmeester[2], Jhon Venegas-Molina[1], Anja David[3], Wilhelm Boland[3] and Marcel Dicke[1]

[1]Laboratory of Entomology, Wageningen University, P.O. Box 16, 6700 AA Wageningen, The Netherlands
[2]Laboratory of Phytopathology, Wageningen University, P.O. Box 16, 6700 AA, Wageningen, The Netherlands
[3]Department of Bioorganic Chemistry, Max Planck Institute for Chemical Ecology, Hans-Knöll-Straße 8, D-07745 Jena, Germany

Involvement of sweet pepper CaLOX2 in jasmonate-dependent induced defence against Western flower thrips

SUPPLEMENTAL INFORMATION

Primers used for (q)RT-PCR.

**For (q)RT-PCR**		
**Gene**	***Fwd-Primer***	***Rev-Primer***
CaLOX2	GCCATTTCTGGATCGGATTA	GCATCAACAGGTGGTGTGAC
CaPINII	CTCGGAATTGTGATACAAGAATTGC	AAGGTACGTACGGCTGCTTCTTTAC
CaPR1	CCTTACGGGGAAAACCTAGC	ACCCTAGCACAACCAAGACG
CaActin	CCCAGATTATGTTTGAGACC	GCAAAGCATAACCCTCATAG
**For (q)RT-PCR post VIGS- To check gene efficiency**		
CaLOX2	GCCATTTCTGGATCGGATTA	GCATCAACAGGTGGTGTGAC
**Primers used to amplify unique region of *CaLOX2* gene**		
CaLOX2	TTCGGATCCCTCAAAGAGGAGCACATTCGT	TTCGGAGCTCTACCAGTGGCATCAACAGGT

Unique region of 282bp of the *CaLOX2* coding region

**Region of CaLOX2 gene used in TRV2 vector to silence the gene down**
CCCTCAAAGAGGAGCACATTCGTGGTCATCTCAATGGAATGACAGTTCAAGAGGCTTTGGATGCAAATAAGCTTTTCATAGTGGATTACCATGAT GTATACCTGCCATTTCTGGATCGGATTAATGCACTTGATGGCCGCAAAGCATATGCAACACGCACCATCTTTTTCTTGTCTAGTTTGGGCACTCTC AAGCCCATTGCCATTGAGCTTAGCCTCCCCCAAACTGGTCCAAGTTCACGATCCAAACGTGTTGTCACACCACCTGTTGATGCCACTGGTA

Induction medium

Induction medium	For 1 litre (g)
K_2HPO_4 (K14)	10.5
KH_2PO_4 (K15)	4.5
$(NH_4)_2SO_4$ (A53)	1
NaCitrate.$2H_2O$ (N20)	0.5
$MgSO_4$ (1M stock)*	1 ml
Glycerol	4 ml
Glucose**	1
Fructose (F028)**	1
MES**	1.95

Set to pH 5.6

* For $MgSO_4$ (1M) stock solution: 49.3 g in 200 ml

** Add after autoclaving

Infiltration medium

Infiltration medium	For 1 litre (g)
MES	1.95
$MgCl_2$ (M003)	2.003
Set to pH 5.4	
Use MQ water	

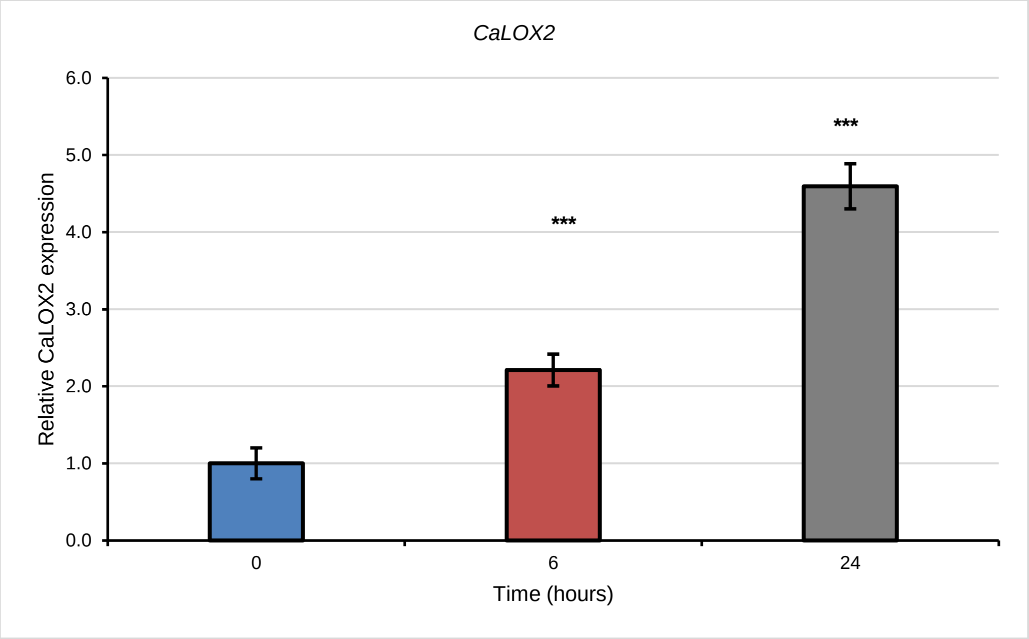

Figure S1. **Expression of *CaLOX2* in GUS-infiltrated samples upon thrips feeding**. The expression level of each gene was normalized to housekeeping gene *CaACTIN*. Data are the means±SE of fourteen biological replicates from two independent experiments.

Asterisks indicate significant differences from control at t=0 (Student's t test), ***p-value < 0.001.
